# National Gay Men’s HIV/AIDS Awareness Day — September 27, 2019

**DOI:** 10.15585/mmwr.mm6837a1

**Published:** 2019-09-20

National Gay Men’s HIV/AIDS Awareness Day, September 27, directs attention to the impact of human immunodeficiency virus (HIV) infection and acquired immunodeficiency syndrome (AIDS) on gay, bisexual, and other men who have sex with men (MSM). In 2017, MSM accounted for 67% of new diagnoses of HIV infection, and MSM who inject drugs an additional 3% (*1*).

To reduce new infections by 90% in 10 years, the Ending the HIV Epidemic national initiative will include efforts to increase preexposure prophylaxis (PrEP) use. From 2014 to 2017, in 20 urban areas, PrEP awareness among MSM increased from 60% to 90%, and PrEP use from 6% to 35% (*2*). However, a report in this issue of *MMWR* shows that, in 2017, a lower percentage of black and Hispanic MSM than white MSM were aware of, had discussed with a health care provider, or had used PrEP (*3*).

CDC supports efforts to reduce HIV infection among MSM, including HIV prevention services that increase diagnosis of HIV infection (https://www.cdc.gov/hiv/group/msm/index.html), support the linkage to and engagement of MSM in care and treatment, and reduce the risk for acquiring and transmitting HIV (https://www.cdc.gov/hiv/group/msm/bmsm.html) (https://www.cdc.gov/msmhealth).

## References

[1] CDC. HIV surveillance report, 2017; vol. 29. Atlanta, GA: US Department of Health and Human Services, CDC; 2018. https://www.cdc.gov/hiv/library/reports/hiv-surveillance.html

[2] Finlayson T, Cha S, Xia M, ; National HIV Behavioral Surveillance Study Group. Changes in HIV preexposure prophylaxis awareness and use among men who have sex with men—20 urban areas, 2014 and 2017. MMWR Morb Mortal Wkly Rep 2019;68:597–603. 10.15585/mmwr.mm6827a1 31298662 PMC6741853

[3] Kanny D, Jeffries WL, Chapin-Bardales J, Racial/ethnic disparities in HIV preexposure prophylaxis among men who have sex with men—23 urban areas, 2017. MMWR Morb Mortal Wkly Rep 2019;68:801–6. 10.15585/mmwr.mm6837a2 PMC6755820 31536484

